# Plenary Speaker Abstracts

**DOI:** 10.1080/24740527.2019.1599267

**Published:** 2019-04-01

## Simplifying Biopsychosocial Pain Management Approaches for Disadvantaged Populations: Reducing Literacy and Cognitive Demands

Beverly E. Thorn

Department of Psychology, The University of Alabama, Tuscaloosa, Alabama, USA

**CONTACT** Beverly E. Thorn bthorn@ua.edu

© 2019 The Author(s). Published with license by Taylor & Francis Group, LLC.

This is an Open Access article distributed under the terms of the Creative Commons Attribution License (http://creativecommons.org/licenses/by/4.0/), which permits unrestricted use, distribution, and reproduction in any medium, provided the original work is properly cited.

Chronic pain is a major public health problem, most recently underscored by the spiraling opioid epidemic, and is unequally borne by individuals with low socioeconomic status (SES). Treatment, when available, is usually biomedical, expensive, and fraught with undesirable side effects. Psychosocial treatments show promise as adjuncts or alternatives, but are usually unavailable to those with low SES, and have not been adapted for patients with low education or literacy levels. Many researchers and practitioners have questioned the feasibility of an approach such as cognitive-behavioral therapy (CBT) for people facing the challenges of limited resources, including low educational attainment and low literacy. CBT requires a certain amount of abstract thinking and problem solving, utilizes written workbooks, handouts and worksheets, and assigns homework, thus requiring more patient effort than passive treatments like taking analgesic medication or receiving biomedical interventions. This presentation will detail a multi-year effort to adapt (simplify) and test the efficacy of patient materials and procedures targeted for highly disadvantaged individuals with the compound risks associated with low-income, including severe and complex pain problems, low-literacy, and minority group membership (African-American). The process of patient-centered adaptation, recruitment, retention, and data collection will be highlighted, and outcomes will be reported. Implementation, durability, and sustainability considerations will be discussed.

Learning Objectives:

1. Understand the reasons for appropriate adaptations of patient materials for individuals with chronic illness.
2. Learn the basics of adaptation of patients materials based on literacy considerations.
3. Understand the basics of biopsychosocial treatment process adaptations to facilitate retention and participation in pain management programs.
4. Learn the evidence base for adapted (simplified) group pain management programs.
5. Understand the challenges of implementation, durability, and sustainability of pain self-management programs.

## Opioid Receptors and Brain Function

Brigitte L. Kieffer

**CONTACT** Brigitte L. Kieffer brigitte.kieffer@douglas.mcgill.ca

© 2019 The Author(s). Published with license by Taylor & Francis Group, LLC.

This is an Open Access article distributed under the terms of the Creative Commons Attribution License (http://creativecommons.org/licenses/by/4.0/), which permits unrestricted use, distribution, and reproduction in any medium, provided the original work is properly cited.

Opiates have been used since thousand years for their remarkable pain-relieving and rewarding properties. Opiates produce their potent effects by activating opioid receptors in the brain, highjacking an endogenous opioid system, which is central to hedonic and mood homeostasis. Recently, revolutions in G protein-coupled receptor research, fascinating developments in basic neuroscience and the rising opioid crisis have propelled opioid receptors back on stage. This presentation will discuss rapidly evolving areas in opioid receptor research for addiction, including the key question of whether we can we kill pain without addiction using mu opiates, and how opioid receptors operate within the neurocircuitry of addiction. Recent work linking mu opioid receptor gene and drug activities to whole-brain functional networks, using by fMRI in mice, will also be presented.

## Clinical Manifestations and Sensitization across Chronic Musculoskeletal Disorders and the Impact on Management

Lars Arendt-Nielsen

Center for Sensory-Motor Interaction, Faculty of Medicine, Aalborg University, Aalborg E, Denmark

**CONTACT** Lars Arendt-Nielsen LAN@hst.aau.dk

© 2019 The Author(s). Published with license by Taylor & Francis Group, LLC.

This is an Open Access article distributed under the terms of the Creative Commons Attribution License (http://creativecommons.org/licenses/by/4.0/), which permits unrestricted use, distribution, and reproduction in any medium, provided the original work is properly cited.

Translating clinical observations to mechanisms and vice versa is notoriously difficult. However, in more recent years human experimental pain assessment tools have been developed to act as proxies for neuronal and perhaps non-neuronal sensitization.

It is generally accepted that pain diagnosis and therapy should be mechanism-based, and hence mechanism-based pain assessment should be sufficiently sensitive and advanced to provide such mechanistic information on sensitization.

As compared with neuropathic pain, little attention has been paid to the fact that chronic musculoskeletal pain disorders may likewise generate neuroplastic changes but is manifested differently from what we see in, e.g. chronic neuropathic pain. Assessing cutaneous allodynia and hyperalgesia is fundamentally easier than evaluating sensory changes in deep musculoskeletal structures (e.g. joints, muscles, bones).

Although it has to be assessed differently in specific tissues for various chronic pain conditions, there are some common underlying mechanistic features, such as central integration (wind-up), centralized facilitation (widespread pain and sensitization), and impaired descending pain inhibition.

As these mechanisms respond differently to, e.g. gabapentinoids, SNRIs, or opioids, mechanistic diagnostic screening tools are important for individualized profiling of patients and hence selecting the drugs which may have the most beneficial effects in patients with different manifestations of sensitization. In recent years, we have also used individualized pain profiling of osteoarthritis patients prior to, e.g. knee replacement surgery and found that specific profiles can predict which patients are the most vulnerable to develop chronic post-operative pain.

Challenges and perspectives to utilize such tools for diagnosis of musculoskeletal sensitization, for management, and ultimately for preventing chronic pain will be highlighted.

